# Case Report: Immune Checkpoint Blockade Plus Interferon-Γ Add-On Antifungal Therapy in the Treatment of Refractory Covid-Associated Pulmonary Aspergillosis and Cerebral Mucormycosis

**DOI:** 10.3389/fimmu.2022.900522

**Published:** 2022-06-01

**Authors:** Alexandra Serris, Amani Ouedrani, Fabrice Uhel, Marianne Gazzano, Vincent Bedarida, Claire Rouzaud, Marie-Elisabeth Bougnoux, Jean-Herlé Raphalen, Sylvain Poirée, Olivier Lambotte, Guillaume Martin-Blondel, Fanny Lanternier

**Affiliations:** ^1^Centre for Infectious Diseases and Tropical Medicine, Hôpital Universitaire Necker-Enfants Malades, Assistance Publique –Hôpitaux de Paris, Université de Paris, Paris, France; ^2^Immunology Laboratory, Hôpital Universitaire Necker-Enfants Malades, Assistance Publique –Hôpitaux de Paris, Université de Paris, Paris, France; ^3^Immunoregulation and Immunopathology, Département Immunologie UMR_S1151 UMR8253 Institut Necker Enfants Malades, Université de Paris, Paris, France; ^4^Intensive Care Medicine, Hôpital Louis Mourier, Assistance Publique –Hôpitaux de Paris, Colombes, France; ^5^Department of Immunologie, Hôpitaux universitaires Pitié Salpêtrière-Charles Foix, Assistance Publique –Hôpitaux de Paris, Paris, France; ^6^Otolaryngology-Head and Neck Surgery Department, Hôpital Lariboisière, Assistance Publique-Hôpitaux de Paris, Paris, France; ^7^Parasitology-Mycology Laboratory, Hôpital Universitaire Necker-Enfants Malades, Assistance Publique –Hôpitaux de Paris, Paris, France; ^8^Intensive Care Medicine, Hôpital Universitaire Necker-Enfants Malades, Assistance Publique –Hôpitaux de Paris, Université de Paris, Paris, France; ^9^Department of Adult radiology, Hôpital Universitaire Necker-Enfants Malades, Assistance Publique –Hôpitaux de Paris, Paris, France; ^10^Service de Médecine Interne Immunologie Clinique, Hôpital Bicêtre, Assistance Publique-Hôpitaux de Paris, Université Paris-Saclay, Le Kremlin Bicêtre, France; ^11^Center for Immunology of Viral, Auto-immune, Hematological and Bacterial diseases (IDMIT/IMVA-HB), UMR1184, Université Paris-Saclay, Inserm, CEA, Le Kremlin Bicêtre, France; ^12^Service des Maladies Infectieuses et Tropicales, CHU de Toulouse, Université Toulouse III, Toulouse, France; ^13^Institut Toulousain des Maladies Infectieuses et Inflammatoires (Infinity) INSERM UMR1291 - CNRS UMR5051 - Université Toulouse III, Toulouse, France; ^14^Molecular Mycology Unit, National Reference Centre for Invasive Mycoses and Antifungals, UMR 2000, Institut Pasteur, CNRS, Université de Paris, Paris, France

**Keywords:** invasive fungal disease, anti-PD 1, cerebral mucormycosis, covid-associated pulmonary aspergillosis, immunotherapy

## Abstract

Invasive fungal diseases (IFD) still cause substantial morbidity and mortality, and new therapeutic approaches are urgently needed. Recent data suggest a benefit of checkpoint inhibitors (ICI). We report the case of a diabetic patient with refractory IFD following a SARSCoV-2 infection treated by ICI and interferon-gamma associated with antifungal treatment.

## Case Report

**Figure 1:**
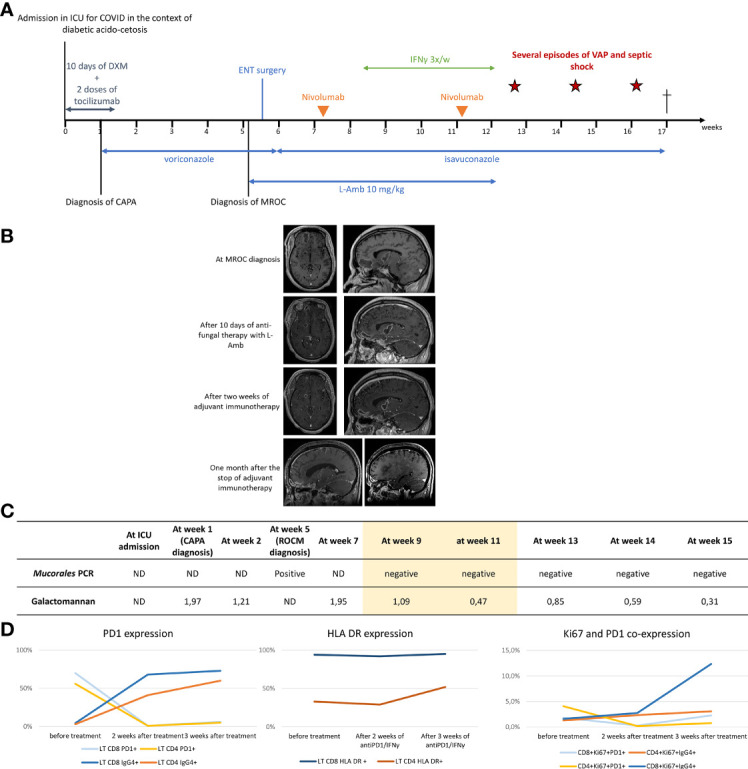
Clinical, radiological and microbiological evolution under treatment. **(A)** Timeline. The cross indicates the death of the patient. **(B)** Evolution of cerebral MRI under treatment. Frontal abscess: 35x15 mm at diagnosis, 32,9 mm immediately after surgical drainage (not shown) 30 x 13 mm after 10 days of antifungal therapy with L-Amb, 24 x 42 mm after two weeks of adjuvant immunotherapy, 14 x 9 mm one week after the stop of adjuvant immunotherapy (not shown) and 9 x 7.2 one month after the stop of adjuvant immunotherapy. lenticulo-capsular abscess: 28 x 14 mm at diagnosis 28 x 17 mm after 10 days of antifungal therapy with L-Amb 24 x 14 mm after two weeks of adjuvant immunotherapy, 17 x 11.5 mm one week after the stop of adjuvant immunotherapy (not shown) and 9.5x7 mm one month after the stop of adjuvant immunotherapy. One morth after the stop of adjuvant immunotherapy, the two initial abscesses had reduced in size. However, shown in the bottom right MRI image, several new cerebral abscesses had appeared. **(C)** Evolution of seric GM and Mucorales PCR under treatment. The yellow color underlines the time under immunotherapy. **(D)** Evolution of PD-1, HLA-DR and Ki67 expression on circulating LT CD4+ and LT CD8+ under treatment. Expression of PD-1,HLA-DR and Ki67 were measured before treatment with nivolumab and 2 and 3 weeks after immunotherapy initiation. After nivolumab injection, PD-1 expression is less, detected as T cells are covered by the anti-PD1 antibody. Cells covered with nivolumab were identified using an anti-IgG4 detection approach. Ki67 has been identified a maker of cellular proliferation and T-cell reinvigoration upon checkpoint blockade (1) As shown in the right graft, the increase in Ki67 expression was most prominent in the IgG4+ versus PD-1+ CD8 and CD4 T celle, i.e., in T cell covered with nivolumab.

A 56 years-old diabetic patient was admitted in February 2021 in ICU for a severe COVID-19 which required immediate mechanical ventilation. The patient received 10 days dexamethasone associated with two doses of tocilizumab according to the local standard of care protocol. One week after ICU admission, COVID-associated pulmonary aspergillosis was diagnosed based on the growth of *Aspergillus flavus* in broncho-alveolar fluid (BAL) culture, positive galactomannan both in BAL (index: 5.9) and in serum (index: 1.9) and new pulmonary infiltrates (**Figure 1A**). Voriconazole was started. A month later, abnormal neurological examination after sedation interruption led to the discovery of two brain abscesses associated with ethmoidal sinusitis (**Figure 1B**). ENT surgery was performed revealing bilateral extensive necrosis of sinonasal mucosa, maxillary and ethmoidal bone. Pathological tissues were respectively removed and drilled until reaching healthy hemorrhagic tissue. A transethmoidal roof procedure allowed for the drainage of the most anterior abscess. *Rhizopus* & *Mucor* PCR was positive in blood as well as in most of per-operative sinus and cerebral samples (**Figure 1C**). Pathology showed aseptate, broad-angled hyphae in the skull bones. Liposomal amphotericin B (L-AmB) was added and voriconazole was switched for isavuconazole when the diagnosis of cerebral mucormycosis was confirmed. Unfortunately, additional debridement was not feasible due to the vicinity of cerebral vessels and brain parenchyma. After ten days of dual antifungal therapy, the patient’s condition worsened as the size of the non-drained abscess increased. Of note, the neutrophils level varied between 4.5 and 12 G/L.

At this step we analyzed by flow cytometry circulating T cells. The total T cell count was normal (1624/mm3) but the CD4/CD8 ratio was below 1 (CD4 = 557/mm^3^). Major lymphocyte subsets were further characterized by their expression of activation/differentiation markers and immune checkpoint molecules. The CD8+ T cell count was increased (CD8 = 938/mm^3^), and 60% of CD8+ T cells co-expressed CD38 and HLA-DR, that are late activation markers. CD4+ T cell expressed also high levels of HLA-DR (33%). Furthermore both CD8+ and CD4+ T cells expressed high levels of programmed cell death-1 (PD-1, 56% and 70%), an immune checkpoint molecule that has been associated with T-cell exhaustion (2). We also measured Aspergillosis and SARS-CoV-2 specific memory T cells using IFN-γ ELISpot assay (3, 4). We detected SARS-CoV-2 but no *Aspergillus* specific T cells in peripheral blood.

Because of the poor prognosis and high percentage of PD-1-expressing circulating CD4 and CD8 T-cells, immunoadjuvant therapy with IFN-γ and nivolumab was started. The patient received 100 mg of interferon-γ SC 3 times a week during a month and 2 IV injections of nivolumab 240 mg (3 mg/kg) four weeks apart. After two weeks of this treatment, the percentage of circulating CD4+ and CD8+ T cells expressing PD-1 substantially decreased, percentage that remained steadily low afterward (**Figure 1D**). After 3 weeks of immunoadjuvant therapy, an increase in CD4 T cells activation (as shown by the rise of CD4 T cells percentage expressing HLA DR) and CD8 T cells proliferative capacity (as indicated by the 4-fold increase of Ki67 expression) were observed (**Figure 1D**). Still, no *Aspergillus* specific T cells could be detected using IFN-γ ELISpot assay. Under combined immunoadjuvant and dual antifungal therapy, the cerebral abscesses decreased in size and serum galactomannan and *Mucorales* PCR turned negative (**Figures 1B, C**).

Unfortunately, after one month the patient developed septic shock following ventilation acquired pneumonia with oliguric renal failure and hepatic cytolysis. L-AmB and IFN γ were stopped and the patient remained under isavuconazole only. Despite several antibiotics and a good monitoring of isavuconazole residual concentration (between 3.1 and 4.2 mg/L) the patient’s condition deteriorated. The size and number of cerebral abscesses increased and the *Mucorales* PCR was found positive in the CSF. After multidisciplinary concertation, palliative care was started, and the patient died 17 weeks after his initial hospitalization.

## Discussion

Rescue therapies are needed for patients not responding to conventional antifungal treatment of severe IFD. Recent advances suggested that immunotherapy might improve the leukocyte immune responses in infectious diseases, notably IFD (5). As several studies have suggested that IFD are associated with an impaired Th1 host immune response, adjunction of recombinant IFNγ has been proposed as a treatment option in patients with poor prognosis IFD (6, 7). Indeed, IFNγ promotes polarization of naïve CD4+ T cells to Th1 cells and exerts positive effects on fungicidal activities of neutrophils *in vitro*. In an open-label case series, it has been shown to partially restore immune function of patients with IFD by enhancing leukocytes activation and promoting their capacity to produce proinflammatory cytokines involved in anti-fungal defense (IL-1, TNFα, IL-17, IL-22) (8).

More recently, it has been shown that,when T cells are exposed to persistent antigens and/or inflammatory signals due to inefficient control of persisting infections, a deterioration of their functions is observed. This state is called “exhaustion” (9). T cell exhaustion has been described in oncology and during chronic infections such as HIV, HCV, and HBV, *Mycobacterium tuberculosis* (10), JC virus infection (11), but also in more acute infections such as malaria (12) and IFD (13). Exhausted T cells are functionally characterized by a loss of IL-2 production, reduced proliferative capacity, reduced cytotoxic capacity, and impaired production of pro-inflammatory cytokines (14). This impaired functional state has been associated with the sustained expression of immune checkpoint molecules, such as PD-1 and cytotoxic T lymphocyte antigen 4 (CTLA-4). It has been shown that modulating these pathways can reverse this dysfunctional state in the context of cancer and infectious diseases (15, 16). Immune checkpoint inhibitors have become major weapons in oncology and standard of care for some metastatic cancers. In infectious diseases, data are much more limited. In animal models of HBV and SIV infections as well as in HIV-infected patients, immune checkpoint blockade resulted in expansion of virus-specific CD4+ and CD8+ T cells with improved functional quality (17, 18). Moreover, some patients with progressive multifocal leukoencephalopathy have been successfully treated with anti-PD-1 therapy (11). In animal models of several IFI, such as aspergillosis, cryptococcosis and histoplasmosis, repetitive administration of anti-PD-1 monoclonal antibodies significantly improved fungal clearance and survival of lethally infected animals (19–21). Therefore, by reversing T cell exhaustion, immune checkpoint blockade represents a therapeutic perspective for cure of deadly IFI.

Two case-reports had previously reported the efficacy of anti-PD-1 therapy combined with IFN-γ in the treatment of mucormycosis (22, 23). In both reports, as in our case, the patients did not respond to conventional antifungal treatment, peripheral T cell showed typical features of exhaustion with increased population of PD-1 expressing T cells and the patient completely or partially responded to immunotherapy with anti-PD-1 and IFN-γ. It is interesting to note that the underlying immune status of the 3 patient was different. Indeed, the first case report concerned a previously healthy 30-year-old woman with extensive abdominal injuries following a terrorist bombing who developed peritoneal invasive mucormycosis. The second case-report involved a highly immunocompromised 51-year-old woman with relapsed acute myeloid leukemia (AML) after hematopoietic stem cell transplant (HSCT) who was diagnosed with a sinus mucormycosis and aspergillosis co-infection (*Lichtheimia ramosa* and *Aspergillus fumigatus*). In our case, the patient can be considered immunocompromised in the context of uncontrolled diabetes mellitus, severe COVID infection treated by corticosteroids and tocilizumab, and sepsis-related immunoparalysis.

Although the patients did not survive in 2 out of the 3 case reports, the initial clinical response obtained with anti-PD-1 associated with IFN-γ, add-on dual antifungal therapy and surgical management, regardless of the patient immune status, might be seen as encouraging. In our case, due to intercurrent nosocomial infections and multi-organs failure, the treatment had to be stopped. Timing is probably of utmost importance in these situations, and it is possible that if this treatment had been initiated sooner, the outcome would have been better. Combination immunotherapy might be considered as salvage treatment of life-threatening IFI unresponsive to conventional therapy. Further studies will be needed to determine its optimal timing and role in the current therapeutic arsenal. Moreover, as PD-1 blockade and IFN therapy reverse T cell exhaustion and enhance leukocytes activation and their capacity to produce proinflammatory cytokines, such an immunotherapy might have more important therapeutic benefits in patients that otherwise do not display quantitative innate immune deficiency such as severe neutropenia.

The use of immune checkpoint inhibitors may however entail severe, notably autoimmune off-the target adverse effects. In a phase Ib randomized study, evaluating 31 adults with sepsis and at least one organ dysfunction, anti-PD-1 (nivolumab) administration was associated with immune-mediated adverse events in 16% of the patients, but did not result in any cytokine storm (24). As patients with IFD are usually particularly fragile and under a lot of concomitant medications, the use of immune checkpoint inhibitor must be carefully balanced and monitored.

## Data Availability Statement

The original contributions presented in the study are included in the article/**Supplementary Material**. Further inquiries can be directed to the corresponding author.

## Ethics Statement

Ethical review and approval was not required for the study on human participants in accordance with the local legislation and institutional requirements. Written informed consent for participation was not required for this study in accordance with the national legislation and the institutional requirements.

## Author Contributions

AS collected the data and wrote the initial manuscript draft. AO and MG did the immunological assays. FL, GM-B and OL coordinated the work and guided the discussion. Other authors (FU, CR, M-EB, SP, VB, J-HR) critically revised the draft and added important intellectual content. All authors participated in review and revisions, approved the final manuscript and are accountable for all aspects of the work and for ensuring that questions related to the accuracy or integrity of any part of the work are appropriately investigated and resolved. All authors had access to and verified the data here presented.

## Conflict of Interest

The authors declare that the research was conducted in the absence of any commercial or financial relationships that could be construed as a potential conflict of interest.

## Publisher’s Note

All claims expressed in this article are solely those of the authors and do not necessarily represent those of their affiliated organizations, or those of the publisher, the editors and the reviewers. Any product that may be evaluated in this article, or claim that may be made by its manufacturer, is not guaranteed or endorsed by the publisher.
