# Supplementary material for: Case Report: Immune Checkpoint Blockade Plus Interferon-Γ Add-On Antifungal Therapy in the Treatment of Refractory Covid-Associated Pulmonary Aspergillosis and Cerebral Mucormycosis
Source: Front Immunol. 2022 Jun 1;13:900522. doi: 10.3389/fimmu.2022.900522 (PMC9199385; doi:10.3389/fimmu.2022.900522)
Supplement: Supplementary file 1 [file Table_1.docx]

**Supplementary**

|  | CD4+CD25+ | CD4+CD38+ | CD4+CD69+ | CD4+HLADR+ | CD8+CD38+ | CD8+CD69+ | CD8+HLADR+ | CD8+HLADR+CD38+ |
| --- | --- | --- | --- | --- | --- | --- | --- | --- |
| Before treatment | 29 % | 28 % | 8 % | 33 % | 87 % | 14 % | 68 % | 60 % |
| 3 weeks after treatment | 26 % | 28 % | 3 % | 52 % | 90 % | 3 % | 73 % | 69 % |

|  | CD4+PD1+ | CD4+TIM3+ | CD4+PD1+TIM3+ | CD4+Ki67+ | CD8+PD1+ | CD8+TIM3+ | CD8+PD1+TIM-3+ | CD8+Ki67+ |
| --- | --- | --- | --- | --- | --- | --- | --- | --- |
| Before treatment | 56 % | 2 % | 1 % | 4 % | 56 % | 2 % | 0 % | 3 % |
| 3 weeks after treatment | 5 % | 3 % | 1 % | 5 % | 2 % | 2 % | 1 % | 12 % |

|  | CD4+CD95+ | CD4+CD95L+ | CD8+CD95+ | CD8+CD95L+ |
| --- | --- | --- | --- | --- |
| Before treatment | 71 % | 6 % | 92 % | 6 % |
| 3 weeks after treatment | 64 % | 2 % | 90 % | 3 % |

**Table S1. Marker expression profiles of CD4 and CD8 subset**
